# Expression Signatures of Cisplatin- and Trametinib-Treated Early-Stage Medaka Melanomas

**DOI:** 10.1534/g3.119.400051

**Published:** 2019-05-17

**Authors:** Barbara Klotz, Susanne Kneitz, Yuan Lu, William Boswell, John Postlethwait, Wesley Warren, Ronald B. Walter, Manfred Schartl

**Affiliations:** * Physiological Chemistry, Biocenter, University of Wuerzburg, 97074 Wuerzburg, Germany; † The Xiphophorus Genetic Stock Center, Department of Chemistry and Biochemistry, 419 Centennial Hall, Texas State University, San Marcos, TX 78666; ‡ Institute of Neuroscience, University of Oregon, Eugene, Oregon, OR 97401; § Genome Sequencing Center, Washington University School of Medicine, St Louis, MO 63108; ** Developmental Biochemistry, University of Wuerzburg, 97074 Wuerzburg, Germany; †† Hagler Institute for Advanced Study and Department of Biology, Texas A&M University, College Station, TX 77843

**Keywords:** Gene expression signature, transgenic medaka model, melanoma, RNA-sequencing, anti-cancer drugs

## Abstract

Small aquarium fish models provide useful systems not only for a better understanding of the molecular basis of many human diseases, but also for first-line screening to identify new drug candidates. For testing new chemical substances, current strategies mostly rely on easy to perform and efficient embryonic screens. Cancer, however, is a disease that develops mainly during juvenile and adult stage. Long-term treatment and the challenge to monitor changes in tumor phenotype make testing of large chemical libraries in juvenile and adult animals cost prohibitive. We hypothesized that changes in the gene expression profile should occur early during anti-tumor treatment, and the disease-associated transcriptional change should provide a reliable readout that can be utilized to evaluate drug-induced effects. For the current study, we used a previously established medaka melanoma model. As proof of principle, we showed that exposure of melanoma developing fish to the drugs cisplatin or trametinib, known cancer therapies, for a period of seven days is sufficient to detect treatment-induced changes in gene expression. By examining whole body transcriptome responses we provide a novel route toward gene panels that recapitulate anti-tumor outcomes thus allowing a screening of thousands of drugs using a whole-body vertebrate model. Our results suggest that using disease-associated transcriptional change to screen therapeutic molecules in small fish model is viable and may be applied to pre-clinical research and development stages in new drug discovery.

Melanoma is one of the most aggressive and most deadly types of skin cancer with an increasing incidence worldwide and a very low cure rate once it has metastasized (Siegel *et al.* 2019). Despite growing awareness of melanoma risk factors, and unique opportunities for early diagnosis, it has become the fifth most frequent cancer in women and the fifth most common cancer in men in the US (https://www.aimatmelanoma.org/about-melanoma/melanoma-stats-facts-and-Figs/, American Cancer Society. “Cancer Facts and Figs 2018”. Atlanta: American Cancer Society; 2018.). New effective treatments like targeted therapies (BRAF- and MEK-inhibitors) are able to increase the one-year progression free survival up to 50% (Larkin *et al.* 2017). However, most patients develop resistance to those drugs after 6-9 months (Long *et al.* 2014). Immunotherapies (CTLA4- and PD1-inhbitor) are effective in 35–60% of melanoma patients, irrespective of the driver mutation (Gide *et al.* 2018; Callahan *et al.* 2018). But for patients with opposing comorbidities or high tumor load and rapid progression, immunotherapy may not be suitable. Also, a considerable percentage of patients suffer from intolerable adverse effects during immunotherapy, forcing them to quit therapy and alter treatment strategies (Collins *et al.* 2016). Importantly, a large number of patients do not benefit from any of the currently available treatment options (Schadendorf *et al.* 2015). Primary or acquired resistance occurs for targeted and immune therapy, leaving a direct clinical need for at least 40–65% of melanoma patients (Gide *et al.* 2018; Callahan *et al.* 2018). Hence, development of effective targeted drugs acting against melanoma growth and progression remains indispensable.

The small aquarium fish medaka (*Oryzias latipes*) is a useful animal model for biomedical research due to several advantages: fish are easy and relatively inexpensive to maintain compared to rodent animal models and their usage is well-accepted to study melanoma development (Patton *et al.* 2010). Another advantage of fish models is their suitability for high-throughput first-line drug screens (Van Rooijen *et al.* 2017). A large number of such screening experiments have been conducted particularly in zebrafish and have established standardized screening technologies by exposing embryos to chemicals and monitoring drug effects (Oxendine *et al.* 2006; White *et al.* 2011; Colanesi *et al.* 2012; Ordas *et al.* 2015). Apart from a stable transgenic medaka line that was used as tumor model for *in vivo* testing of a known anticancer drug (Matsuzaki *et al.* 2013), efficient systems to monitor the influence of drugs on melanoma development and progression in free swimming larvae or adult fish are still missing. Major obstacles for developing such screens are the difficulties to evaluate possible drug-induced effects on tumor growth in large numbers of animals and the long times, generally weeks to months, for such changes to become visible. Cancer is a systemic disease and develops generally at much later stages than those accessible in embryonic screen protocols. Earlier study has exhibited that melanoma development trajectory is determined by early stage genetic changes (Schartl *et al.* 2015).

We hypothesize that drug treatments should lead to early changes of gene expression levels long before any alterations of the tumor phenotype (tumor load, invasion and metastasis) become obvious.

To test this hypothesis, we used the transgenic medaka melanoma model. In these fish, malignant pigment cell tumors develop due to expression of the *xmrk* driver oncogene, which is a constitutively activated mutant version of the epidermal growth factor (Egf) receptor under control of the medaka melanocyte-specific *mitfa* promoter (Schartl *et al.* 2010). In these transgenic fish, melanoma development initiates in larvae and progresses further in juvenile and adult stages. The tumors are highly similar on the histopathological and molecular level to the native *Xiphophorus* melanoma system from which the oncogene is derived. Importantly, gene expression studies showed that these *xmrk*-induced fish melanomas are comparable to human tumors (Schartl *et al.* 2012; Schartl *et al.* 2015; Klotz *et al.* 2018; Lu *et al.* 2018) and thus provide suitable models for experimental studies. Using melanoma-effective drugs cisplatin and trametinib, we performed short-term exposures of juvenile medaka with developing melanoma, and compared the modulated gene expression profiles to similarly treated healthy wild-type medaka. Herein, we detail changes in gene expression that can be monitored from whole-fish total RNA extraction after short-term drug exposures. Our previous study showed that transcriptional changes induced by a disease-driving oncogene (*i.e.*, *xmrk*) could collectively serve as a transcriptional phenotype for disease diagnosis, and can be used for pilot drug screening. However, different classes of medicine (*e.g.*, kinase inhibitor, hormone) act in different mechanisms and may lead to different transcriptional changes. Therefore, this manuscript aims to test whether small molecule treatment can lead early to transcriptional changes. These results can be used to establish treatment specific transcriptional disease signatures (TTDS) to develop high throughput chemical screens for substances with potential for melanoma therapeutics.

## Material and Methods

### Experimental Animals and fish maintenance

Wild-type (wt, inbred strain Carbio) and transgenic (tg) medaka of the Tg(*mitf:xmrk*) strain on the Carbio genetic background (Schartl *et al.* 2010) were used for this study. Fish were maintained under standard conditions in the aquarium facility of the Biocenter at the University of Wuerzburg according to local animal welfare laws and guidelines and with the authorization (55.2 – 2531.01 – 40/14) of the Veterinary Office of the District Government of Lower Franconia, Germany, in accordance with German Animal Protection Laws.

### Compound treatment

Cisplatin (Merck KGaA, Darmstadt, Germany) and trametinib (Selleck Chemicals, Houston, USA) and their corresponding solvents were used for treatment. Cisplatin was administered at a concentration of 70 μM (solvent 0.9% NaCl), and trametinib was used at a concentration of 20 nM (solvent 1% DMSO). Fish between 3-5 weeks of age and a standard length of 10 ± 2 mm were exposed to these substances over seven days. Fish were divided into control (solvent-treated) and drug-treated groups and maintained in small petri dishes in a volume of 20 ml conditioned tank water at 23° at 12 hr/12 hr light/dark cycle. After daily feeding with *Artemia naupliae*, the water was changed and drugs or solvents alone were freshly added. For RNA sequencing, 10 transgenic (tg) and 5 wild-type (wt) fish each treated with cisplatin or trametinib and 5 wt and 5 tg fish that were treated with solvent were used.

### RNA-sequencing (RNA-seq)

Total RNA from the whole fish body was isolated using TRIzol Reagent (Thermo Fisher Scientific, Waltham, USA) according to the supplier’s recommendation. RNA Integrity Number (RIN) was assessed using an Agilent 2100 Electrophoresis Bioanalyzer Instrument G2939A (Agilent Technologies, Böblingen, Germany). Only samples with RIN values > 8 were used for sequencing. RNA sequencing libraries were produced according to the standard Illumina mRNA library preparation protocol (www.illumina.com; Illumina Inc., San Diego, CA, U.S.A.) and sequenced at 150 bp paired end reads with a sequencing depth of approx. 72 million reads per sample.

### RNA-seq validation by quantitative real-time PCR (qRT-PCR)

Whole treated and solvent-treated wt and tg three to five week old fish (n= 5 or 10 in each group) were used to extract total RNA using TRIzol Reagent (Thermo Fisher Scientific, Waltham, USA) according to the supplier’s recommendation. The RNA (2 μM) was treated with DNase and transcribed into cDNA using the RevertAid First Strand cDNA Synthesis kit (Thermo Fisher Scientific, Waltham, USA) with random hexamer primers in accordance with the manufacturer’s instructions. This cDNA was used as a template for qRT-PCR in a 25 μl volume SYBR Green reagent containing mastermix and was analyzed in triplicate. Amplifications were carried out in a Mastercycler ep realplex2 (Eppendorf, Hamburg, Germany) with cycling parameters as follows: 95° for 5 min, followed by 40 cycles of 95° for 30 s, 60° for 30 s and 72° for 20 s. For qRT-PCR-Primer sequences, see Table S1. Quantitative RT-PCR data were quantified by the delta Ct method (Simpson *et al.* 2000) and the expression of each gene of interest was normalized to the housekeeping gene *ef1a1* (elongation factor 1 alpha 1). Statistical significance of different mRNA expression levels was checked using Wilcoxon-Mann-Whitney *U*-test or Student’s *t*-test depending on the size and distribution of the samples. Student’s *t*-test was used for normally distributed data with a sample size larger than nine. Data without normal distribution or with a sample size less than nine were analyzed using the non-parametric Wilcoxon-Mann-Whitney *U*-test. A selection of differentially expressed genes was validated by qRT-PCR and confirmed the direction of regulation from the RNA-seq data (Table S2).

### Bioinformatics, statistical analysis and differential gene expression

Paired end sequences were trimmed using Trimmomatic (-phred33) with the TruSeq3-PE.fa as adapter file. Sequences were aligned to the medaka genome (ASM223467v1) using the STAR RNA-seq aligner (Dobin *et al.* 2013) and the genes were quantified by RSEM (Li and Dewey 2011). Differential expression was calculated by the R/Bioconductor package ‘DESeq2’ (Love *et al.* 2014). For correspondence analysis, the R/Bioconductor package ‘made4’ was used. Genes were considered to be differentially expressed in comparison to the corresponding solvent-treated wt or tg fish, if they had a log2 fold change (log2FC) <-1 or > +1 and a p-value < 0.05.

### Pathway and transcription regulator enrichment

First, we focused on genes that were up- and downregulated (log2FC <-1 or > +1, p-value < 0.05) in one of the following groups: a) in both cisplatin-treated wt and tg fish; b) in cisplatin-treated wt fish *vs.* solvent-(NaCl)-treated wt fish; c) in cisplatin-treated tg fish *vs.* solvent-(NaCl)-treated tg fish; d) expressed in both trametinib-treated wt and tg fish; e) in trametinib-treated wt fish *vs.* solvent-(DMSO)-treated wt fish and f) in trametinib-treated tg fish *vs.* DMSO-treated tg fish. To enable further functional analysis of anti-melanoma drug-treated wt and tg whole body transcriptomes, differentially expressed medaka genes were matched to their human orthologs using Ensembl BioMart (http://www.ensembl.org/biomart/martview/662764dc0a1ef355f59d8c648d5196be), the DAVID Gene Accession Conversion Tool (https://david.ncifcrf.gov/conversion.jsp) and manual annotation after BLASTN searches https://www.ensembl.org/Multi/Tools/Blast?db=core) for genes that were not annotated in the medaka genome. WebGestalt (http://www.webgestalt.org/2013/option.php) and DAVID (https://david.ncifcrf.gov/) functional enrichment analysis web tools were used for functional classification using default settings. Some of the over-represented KEGG pathways in Trametinib-treated tg samples were selected and colored with the Search&Color Pathway tool (https://www.genome.jp/kegg/tool/map_pathway2.html).

### Data availability

All data necessary to confirm the conclusions of the current article are represented fully within this manuscript and the supplemental material. Supplemental material available at FigShare: https://doi.org/10.25387/g3.8075777.

## Results and Discussion

The intention of the current study was to uncover treatment-induced gene expression changes in the tg medaka melanoma model (Schartl *et al.* 2010) compared to healthy fish. Two established anti-cancer drugs, cisplatin and trametinib, were administered to juvenile medaka over seven days to investigate drug-specific effects on single gene expression patterns and genetic pathways compared to solvent-treated control fish. The MEK-inhibitor trametinib and the cytostatic drug cisplatin, which inhibits DNA replication, differ in their mode of molecular action. Activation of the Ras-Raf-MEK-ERK (MAPK) pathway is a common feature of most human melanomas and is also the driver signaling pathway in the tg melanoma medaka (Meierjohann and Schartl 2006; Wellbrock and Arozarena 2016). Both substances are approved for clinical application. Cisplatin is effective against different types of cancers, including melanoma. It is a platinum-based chemotherapeutic drug, which crosslinks DNA and thereby interferes with DNA replication and mitosis and induces pro-apoptotic effects (Wang and Lippard 2005; Kelland 2007).

Whole-body gene expression profiles from cisplatin (70 μM) and trametinib (20 nM) treated juvenile (3-5 weeks old) healthy (wt) and early-stage melanoma (tg) fish were compared to solvent-treated fish (wt and tg). Differentially expressed genes were used to define specific signatures of cisplatin- or trametinib-treated wt and tg fish compared to controls. Earlier gene expression studies revealed the feasibility to extract melanoma-specific gene expression patterns from transcriptomic data based on whole fish body RNA isolates and the response of the juvenile fish (`host response’) to the initial melanoma stage (Schartl *et al.* 2015; Klotz *et al.* 2018). In this study RNA based gene expression signatures of similar and different patterns for gene expression in treated wt and tg medaka were established. `Similarities` between treated wt and tg fish were interpreted as global drug-induced effects that point to drug-induced side effects, whereas `differences` between treated wt and tg fish were considered to identify drug-specific response in disease (drug-specific treatment signature).

### Cisplatin

**Figure 1:**
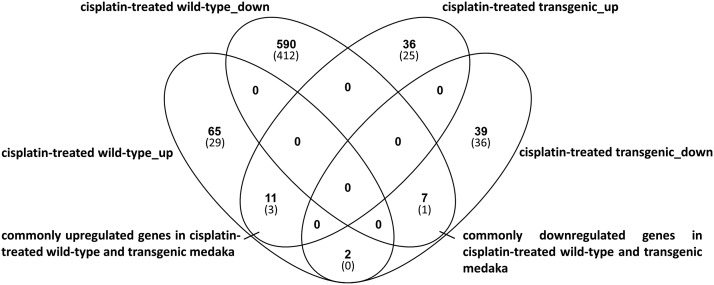
The Venn-diagram represents differentially expressed genes (in brackets: human orthologs) in cisplatin-treated wild-type and transgenic medaka.

The differential expression analysis of cisplatin-treated medaka compared to solvent-(NaCl)-treated medaka identified 655 genes that were differentially expressed comparing treated to untreated wt fish (n = 5) and 75 genes that were differentially expressed comparing treated to untreated tg fish (n = 10). We identified 65 up- and 590 downregulated genes exclusively in wt fish and 36 up- and 39 downregulated genes exclusively in tg medaka (Figure 1). We matched differentially regulated medaka genes to their human orthologs to enable further functional analysis. Of the 655 differentially regulated genes in wt medaka, 441 (29 upregulated, 412 downregulated) matched to human gene annotation and 61 (25 up, 36 down) of 75 differentially regulated genes in tg fish matched human orthologs (Figure 1).

### Commonly regulated genes in cisplatin-treated wt and tg fish

Commonly up- and down-regulated genes in cisplatin-treated wt and tg fish are considered as general drug-induced effects representing possibly unwanted or adverse side effects. We identified 11 commonly upregulated and seven commonly downregulated genes. Three (*fabp1*, *mdm2*, *slc19a3*) of 11 commonly upregulated and one (*kcnj1*) of seven commonly downregulated genes in juvenile cisplatin-treated wt and tg medaka corresponded to human orthologs (Figure 1), and information about their function is available to allow for biological inference of cancer influences.

Fatty acid binding protein 1 (*fabp1*) is highly expressed in hepatocytes but is also found in other cell types and tissues (Iseki *et al.* 1990; Pelsers *et al.* 2003). FABP1 is involved in the cellular uptake, transport and metabolism of fatty acids as well as in regulation of gene expression and cell differentiation (Wang *et al.* 2015). Cisplatin-injected sv129 mice showed increased *fabp1* mRNA expression levels three days after injection in kidney tissue compared to saline-treated mice (Negishi *et al.* 2007). At the protein level, cisplatin treatment of rat primary hepatocytes over 24 hr induced FABP1 expression (Cho *et al.* 2012). These findings are in accordance with the enhanced *fabp1* expression we found in cisplatin-treated medaka. Human liver cells transfected with FABP1 cDNA showed higher resistance toward Acetaminophen-induced hepatotoxicity compared to empty vector transfected cells (Gong *et al.* 2014) and this result was explained by an antioxidant function of FABP1 expressing cells. The cisplatin-induced increase of *fabp1* expression in wt and tg medaka may be also an organismal strategy to fight the cisplatin-triggered increase of reactive oxygen species levels.

Mdm2 (encoded by *mdm2*) is an E3 ubiquitin ligase localized in the nucleus that acts as an inhibitor of p53 (tumor protein p53) through the induction of p53 protein degradation. DNA-damaging agents like cisplatin activate p53, which in turn upregulates MDM2 (Moll and Petrenko 2003; Basu and Krishnamurthy 2010). This finding is reflected in cisplatin-treated murine testicular cancer cell lines showing increased expression levels of *Mdm2* mRNA (Di Pietro *et al.* 2012). We interpret the increased expression of *mdm2* as a general cisplatin-induced DNA damage response.

*slc19a3* (solute carrier family 19, member 3) encodes a thiamine transporter (Ganapathy *et al.* 2004) and therefore its upregulation might reflect its function as transporter for drug absorption, elimination or possibly mediating chemosensitivity.

The *kcnj1* (potassium voltage-gated channel subfamily J member 1) gene was identified as the only commonly downregulated gene in cisplatin-treated wt and tg fish. This ATP-dependent potassium channel is important for the homeostasis of potassium channels and is predominantly found in kidney tissues of rats and humans (Nüsing *et al.* 2005; Welling and Ho 2009). It is also expressed in cells and organs associated with osmoregulation in developing zebrafish embryos (Abbas *et al.* 2011) but has not been reported so far to be regulated in response to cisplatin treatment.

Besides the commonly regulated genes in wt and tg medaka, which represent the side effects of the drug, we also found genes that were only affected by the drug treatment in wt fish (see Supplemental Note 1).

### Cisplatin-induced specific effects in juvenile tg medaka

Many of the enriched GO terms in cisplatin-treated tg melanoma medaka compared to vehicle-treated tg melanoma controls are associated with pigmentation and cellular transport activity. All GO terms comprise one of the following three groups of upregulated genes (Table S3): alkaline phosphatases (*alpi*, *alpp*, *alppl2* (alkaline phosphatase, intestinal, placental and placental-like 2)), members of the solute carrier family (*slc3a1*, *slc6a19*, *slc15a1*, *slc26a6)* as well as the *bloc1* genes (*bloc1s6* (biogenesis of lysosomal organelles complex-1, subunit 6, pallidin), *bloc1s4* (biogenesis of lysosomal organelles complex-1, subunit 4, cappuccino), *bloc1s1* (biogenesis of lysosomal organelles complex-1, subunit 1)). These *bloc1* genes encode proteins forming subunits of the cytosolic protein complex BLOC-1 (biogenesis of lysosome‐related organelles complex-1), which plays an important role in the formation of specialized organelles of the endosomal-lysosomal system for example melanosomes (Starcevic and Dell’angelica 2004). This result is in accordance with the tumorigenic phenotype of the tg medaka caused by the expression of *xmrk* triggering hyperpigmentation of the skin already some days after hatching and the formation of pigment cell tumors as described earlier (Schartl *et al.* 2015; Klotz *et al.* 2018). We did not observe any phenotypic changes in the cisplatin-treated tg medaka (data not shown). It is interesting to note that several of the genes and enriched GO terms that are affected by the treatment are part of the melanomatous fish specific expression profile identified in two earlier studies (Schartl *et al.* 2015; Klotz *et al.* 2018). In addition, a set of upregulated genes and enriched GO terms appear as “early cisplatin-induced gene expression change”. Untreated tg fish from the two earlier studies showed an upregulation of *tyrp1* (tyrosinase-related protein 1), *pmel* (premelanosome protein) or *tyr* (tyrosinase), all known as typical melanin synthesis and pigmentation associated genes (Schartl *et al.* 2015; Ainger *et al.* 2017; Klotz *et al.* 2018). These genes were not upregulated in the cisplatin-treated tg fish compared to solvent-treated tg fish, but we detected an enhanced expression of the *BLOC* genes indicating a drug effect on melanoma on the gene expression level. Additionally, the cisplatin-treated tg fish showed an enrichment of the GO terms `melanosome organization’ (GO:0032438) and `pigment granule organization` (GO:0048753) displaying potentially cisplatin-affected pigmentation-associated transformation processes in the treated medaka (Table S3).

The upregulated genes of the solute carrier family in many enriched GO terms (*e.g.*, `secondary active transmembrane transporter activity` (GO:0015291) or `solute:cation symporter activity` (GO:0015294)) (Table S3) are of special interest because they function as membrane influx or also bi-directional transporters mediating the uptake, distribution, metabolization and elimination of substrates like glucose, amino acids, metals and most importantly (anti-cancer) drugs (He *et al.* 2009; Li and Shu 2014). Solute carriers have profound influence on drug efficacy. We noted upregulated expression of the genes *slc3a1* (solute carrier family 3 (cystine, dibasic and neutral amino acid transporters, activator of cystine, dibasic and neutral amino acid transport), member 1), *slc6a19* (solute carrier family 6 (neutral amino acid transporter), member 19), *slc26a6* (solute carrier family 26, member 6) and *slc15a1* (solute carrier family 15 (oligopeptide transporter), member 1) in the cisplatin-treated melanoma medaka. We assume that the upregulated expression of these genes is a result of absorbing, metabolizing and eliminating processes due to cisplatin treatment (Li and Shu 2014).

Among the downregulated genes, we identified an enrichment of 27 GO terms including many cell cycle associated GO terms (*e.g.*, `mitotic cell cycle` (GO:0000278), `cell cycle process` (GO:0022402)) as well as GO terms related to cell biology and cellular processes in general (*e.g.*, `cytoskeletal part` (GO:0044430), `cytosol`(GO:0005829)) (Table S4).

*racgap1* (Rac GTPase activating protein 1), a member of the family of Rho GTPase-activating proteins (Touré *et al.* 1998; Jantsch-Plunger *et al.* 2000), is one of the downregulated genes included in the enriched GO terms. It is important for the induction and regulation of cytokinesis, cellular growth and differentiation but also for transformation and metastasis (Hirose *et al.* 2001; Kitamura *et al.* 2001; Mi *et al.* 2016). Its expression is elevated in several cancer types like breast (Pliarchopoulou *et al.* 2013), gastric (Saigusa *et al.* 2015) or lung cancer (Liang *et al.* 2013) where high *RACGAP1* expression is accompanied by greater aggressiveness and invasion of tumors as well as poor prognosis for patients. These findings provide strong evidence for a cisplatin-caused suppression of the proliferative and transcriptionally active state of the melanoma tumor in tg fish samples.

Ataxia telangiectasia mutated (*atm*) was also downregulated. A mutated version of the *ATM* gene causes ataxia telangiectasia, a disease causing increased susceptibility to cancer (Savitsky *et al.* 1995). *ATM* encodes a kinase important for the repair of DNA double-strand breaks (Shiloh 2003). It was reported that low ATM expression and poor survival rate correlate in cancer patients (Knappskog *et al.* 2012; Feng *et al.* 2015; Petersen *et al.* 2017). The downregulation of *atm* in the cisplatin-treated tg samples would result in a compromised repair of DNA leading to an already earlier described increase of cisplatin-induced apoptosis (Colton *et al.* 2006).

Two downregulated genes, *gmppb* (GDP-mannose pyrophosphorylase B) and *gmds* (GDP-mannose 4,6-dehydratase) are members of the enriched KEGG pathways `fructose and mannose metabolism` (ID:00051) and `amino sugar and nucleotide sugar metabolism` (ID:01100) (Table S5). The Gmppb protein (Noda *et al.* 2003) is important for the production of N-linked oligosaccharides (Ning and Elbein 2000) and the inhibition of the N-linked oligosaccharides leads to reduced invasion of tumor cells *in vitro* (Dennis 1991). Gmds catalyzes the first step of the *de-novo* synthesis of GDP-fucose, which is an essential component of cellular fucosylation (Becker and Lowe 2003). Deregulated fucosylation is associated with different cancer types (*e.g.*, hepatocellular or ovarian carcinoma) (Noda *et al.* 2003; Escrevente *et al.* 2006; Wei *et al.* 2018). GDMS was upregulated in tissues from lung adenocarcinoma patients and its knockdown induced cell cycle arrest and apoptosis in human lung adenocarcinoma cell lines (A549, H1299) as well as an inhibited tumor growth in a xenograft mice model of lung adenocarcinoma (Wei *et al.* 2018). Both enriched KEGG pathways are linked to carbohydrate metabolism and its alteration is typical for cancer cells requiring high energy production for their uncontrolled cellular proliferation. Downregulation of these two-carbohydrate metabolism related genes proposes that cisplatin treatment of melanoma fish has an inhibiting effect on carbohydrate metabolism and its energy producing steps and therefore may suppress uncontrolled cell growth. This may be in concert with the upregulation of fatty acid binding protein as a shift from carbohydrate to lipid metabolism as a primary energy source.

We also observed cisplatin-induced expression changes of members of the alkaline phosphatase family (*alpi*, *alpp*, *alppl2*). Several studies indicate elevated alkaline phosphatase levels as evidence for hepatobiliary diseases (Moss 1989; Giannini *et al.* 2005; Lala and Minter 2018). The upregulation of *alpi*, *alpp* and *alppl2* can be a direct result of the metabolization of the absorbed drug cisplatin in the tg medaka. This could be interpreted as adverse effects of the drug treatment.

### Trametinib

**Figure 2:**
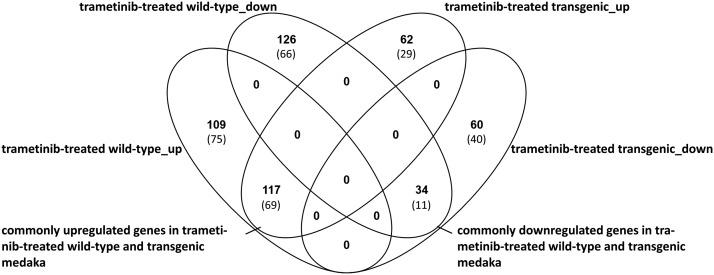
The Venn-diagram represents differentially expressed genes (in brackets: human orthologs) in trametinib-treated wild-type and transgenic medaka.

The differential expression analysis of trametinib-treated medaka compared to solvent-(DMSO)-treated medaka resulted in 109 up- and 126 downregulated genes exclusively in wt fish (n = 5) and in 125 differentially expressed genes exclusively in tg fish (62 upregulated, 60 downregulated) (n = 10). A total of 141 of 235 differentially expressed genes in trametinib-treated wt fish matched to human orthologs (75 upregulated, 66 downregulated). In tg medaka, 29 of 62 upregulated genes and 40 of 60 downregulated genes matched to their human gene orthologs (Figure 2).

### Commonly expressed genes in trametinib-treated wt and tg fish

Comparing trametinib-induced specific expression changes in wt and tg medaka, 69 of the 117 commonly upregulated genes and 17 of the 34 commonly downregulated genes matched to human orthologs (Figure 2). Trametinib-induced effects common to both wt and tg fish included upregulation of immune system associated genes and genes related to cellular transporter activity, as well as oxidoreductase activity (Table S6). Upregulated immune associated genes could be a general response of the fish to the trametinib-induced pro-inflammatory and pro-apoptotic effects described by Lulli *et al.* (2017). An attempt to eliminate this drug may be reflected by the enhanced expression of the drug metabolizing enzymes *cyp24a1* (cytochrome P450, family 24, subfamily A, polypeptide 1) and *cyp21a2* (cytochrome P450, family 21, subfamily A, polypeptide 2). The cytochrome P450 enzyme family plays a crucial role in the biotransformation and elimination of drugs (Ogu and Maxa 2000; Mittal *et al.* 2015). Furthermore, trametinib also induced complement system genes, a hallmark of inflammatory processes (Frank and Fries 1991; Kirschfink 1997; Markiewski and Lambris 2007) that was not observed in the DMSO-treated wt and tg medaka. The activation of the complement system, which is normally triggered by pathogens to combat infections (Dunkelberger and Song 2010), may be involved in eliminating damaged and apoptotic cells (Noris and Remuzzi 2013). Our data suggest that trametinib activates inflammatory and immune system associated processes in wt and tg medaka.

Trametinib induced a downregulation of several genes with GO terms `regulation of tissue remodeling` (GO:0034103), `motor neuron axon guidance` (GO:0008045) or `regulation of cell-substrate junction assembly` (GO:0090109) in wt and tg fish (Table S7). These include *etv4* (ets variant 4), *grem1* (gremlin 1) and *hrg* (Histidine Rich Glycoprotein). The *etv4* gene encodes a member of the ETS transcription factor family, which is involved in organ development, cell proliferation and tumorigenesis (Chotteau-Lelièvre *et al.* 1997; Dumortier *et al.* 2018). Increased expression of *ETV4* was reported in several types of cancer (*e.g.*, breast (Yuan *et al.* 2014; Dumortier *et al.* 2018) and prostate cancer (Pellecchia *et al.* 2012; Qi *et al.* 2015) and esophageal squamous cell carcinoma (Qi *et al.* 2015)). The trametinib-induced downregulation of *etv4* in tg fish is in accordance with the finding that trametinib treatment leads to a reduced expression of *ETV4* in pancreatic cancer cell lines (Wang *et al.* 2017). Trametinib treatment resulted in a much stronger downregulation of *etv4* in melanoma-bearing tg medaka than in wt fish. We conclude that the trametinib-induced downregulation of *etv4* functions inhibits cell proliferation in wt and tg fish, because it has been shown that decreased *ETV4* expression in malignant cells is accompanied by reduced cell growth and migration (Pellecchia *et al.* 2012).

The member of the BMP (bone morphogenic protein) antagonist family *grem1* (gremlin 1) (Church *et al.* 2015) is also downregulated in trametinib-treated wt and tg medaka. Grem1 is involved in embryonic development (Costello *et al.* 2010) but it was also found to be expressed in stromal cells of human tumorigenic tissues contributing to a tumor microenvironment regulated growth and invasion of cancer (Sneddon *et al.* 2006). Moreover, increased *GREM1* mRNA levels were observed in several different cancer types (*e.g.*, lung, colon, breast or pancreas) (Namkoong *et al.* 2006). With the genes *grem1* and *etv4*, we identified two genes involved in developmental and carcinogenic processes and our data show that trametinib targets the expression of such potential carcinogenic candidate genes irrespective of whether its administration occurred in a tumor situation or not, indicating that this drug exerts an inhibitory influence on developmental and proliferative states in cells and organisms.

Besides the commonly regulated genes in wt and tg medaka we also found genes that were only affected by the drug treatment in wt fish (see Supplemental Note 2).

### Trametinib-induced effects on juvenile tg medaka

Trametinib treatment changed the expression profile of the tg fish compared to the solvent-treated tg medaka as illustrated by a high number of enriched GO terms and KEGG pathways (Table S8, S9, Table S10, Table S11). Genes of the enriched `MAPK (mitogen-activated protein kinase) signaling pathway` are up- or down-regulated. This finding is of particular interest because: 1) an activated MAPK signaling pathway is often observed in human cancers including melanoma (Davies *et al.* 2002; Ohren *et al.* 2004; Dhillon *et al.* 2007; Inamdar *et al.* 2010) and 2) the trametinib-induced inhibition of the MEK1/2 protein kinases leading to a reduction of downstream MAPK signaling and cell growth of tumor cells can be seen as evidence for the drug effectiveness in the tg melanoma fish. Upon trametinib treatment *gadd45g* (growth arrest and DNA-damage-inducible, gamma) and *mknk2* (MAP kinase interacting serine/threonine kinase 2) are upregulated, while *dusp4*, *dusp6* (dual specificity phosphatase 4, 6) and *nr4a1* (*nur77*, nuclear receptor subfamily 4, group A, member 1) are downregulated in the tg fish. The tumor suppressor gene *gadd45g* is a member of the *GADD45* family and plays an important role in cell cycle arrest (Ying *et al.* 2005) and its overexpression has an inhibitory function on the proliferation of transformed cells. Downregulation of *gadd45g* has been described in many different tumor cell lines (Zhan *et al.* 1994; Vairapandi *et al.* 2002; Ying *et al.* 2005; Zerbini and Libermann 2005). The trametinib-treated tg samples showed an enhanced *gadd45g* expression level implying a trametinib-induced transition from a melanogenic expression profile toward a less cancerogenic and more normal expression profile. The melanogenic profile is still visible by the expression of *dusp4*, *dusp6* and *nr4a1*, which is, however, strongly diminished, as expected if the drug were normalizing the transcriptomic disease phenotype. Interestingly, Tamura *et al.* (2012) described an enhanced expression of the GADD45 proteins due to anti-cancer drugs like Genistein or Trichostatin A along with a growth arrest in different tumor cell lines. These findings are in line with the increased expression of *gadd45g* in the trametinib-treated medaka in the current study. Besides an upregulation, the MAPK cascade in the trametinib-treated samples also shows a number of downregulated genes, thus providing evidence for the efficacy of the trametinib treatment: we identified *flt4* (fms-related tyrosine kinase 4), *nr4a1* and members of the *dusp* family as downregulated genes within the MAPK signaling pathway, which are located at main nodes or directly influencing important members of this signaling cascade. Flt4, a tyrosine kinase receptor for the ligand VEGF-C (vascular endothelial growth factors C), is positioned at the top of the MAPK signaling cascade and it is known to be expressed on high levels in several human cancer types including non-small cell lung cancer tissues from patients (Li *et al.* 2006), advanced-stage specimens of prostate cancer (Kaushal *et al.* 2005), head and neck tumors (Neuchrist *et al.* 2003) or sera samples from patients suffering from metastatic melanoma (Mouawad *et al.* 2009). These reports indicate a connection between enhanced *flt4* expression and tumor development and progression. Furthermore, *FLT4* is involved in sprouting angiogenesis and it is highly expressed in the microvasculature of tumors and wounds (Valtola *et al.* 1999; Paavonen *et al.* 2000). Antibody-mediated blocking of FLT4 signaling leads to reduced blood vessel sprouting, vessel branching, and endothelial cell growth in mouse angiogenesis models (Tammela *et al.* 2008). Transferring these findings to our data, the trametinib-induced decrease of *flt4* expression might result in decreased development of tumor-supporting blood vessels in trametinib-treated tg medaka.

Dusp4 and Dusp6 dephosphorylate kinases leading to an inactivation of the MAPK family members ERK, JNK and p38, which are closely associated with processes of cellular proliferation and differentiation, and therefore, act as negative regulators of MAPK signaling (Clark *et al.* 2008; Huang and Tan 2012). The downregulation of both genes in our treatments may be reconciled by the finding that BRAF or MEK inhibition significantly reduces expression of *dusp4*, *dusp6*, *spry2* and *spry4* (Sprouty RTK Signaling Antagonist 2, 4) (Pratilas *et al.* 2009). Beside the *dusp* genes, also a reduced expression of *spry4* was observed in the trametinib-treated fish. The Ras/ERK pathway induces expression of SPRY and dysregulation of *SPRY* genes occurs in many tumor types *e.g.*, cancer of the breast (Faratian *et al.* 2011; Vanas *et al.* 2014) and liver (Sirivatanauksorn *et al.* 2012). Reports also describe the upregulation of *SPRY2* expression in melanoma cells with mutated *B-Raf* or *N-Ras* compared to melanomas with wild-type *B-Raf* probably due to theenhanced expression or activity of p-ERK in these cells (Tsavachidou *et al.* 2004; Bloethner *et al.* 2005). Therefore, the downregulation of *spry4* in trametinib-treated tg medaka could be a direct consequence of the reduced expression of *flt4* localized upstream in the MAPK signaling cascade.

The nuclear receptor *nr4a1* is an immediate-early response gene involved in cell proliferation and differentiation. We interpret the downregulation of *nr4a1* in trametinib-treated samples to provide further evidence for the transition of a transformed phenotype toward a less malignant melanoma phenotype after trametinib treatment because high *nr4a1* expression has often been detected in tumorigenic tissues and cells or solid tumors (Cho *et al.* 2007; Cho *et al.* 2010; Lee *et al.* 2010; Lee *et al.* 2014; To *et al.* 2014). Ectopic expression of *NR4A1* promotes cell cycle progression and proliferation in lung cancer cells (Kolluri *et al.* 2003), at the same time *NR4A1* repression has an inhibitory effect on the transition phenotype of several cancer cell types by promoting the induction of apoptosis (Ke *et al.* 2004). However, stable knockdown of *NR4A1* by RNA interference conferred resistance of melanoma cells to apoptosis induced by chemotherapeutic drugs (*e.g.*, Cisplatin) (Yu *et al.* 2007). Therefore, the reduced *nr4a1* expression as a result of the trametinib treatment could also be interpreted as adverse effect of the drug treatment in the tg medaka. But there is also a report that the inhibition of MAPK signaling in melanoma cells either by BRAF knockdown or by the usage of the MEK1/2 specific inhibitors UO126 and PD98059 leads to a clear reduction of the *NR4A1* expression (Smith *et al.* 2011).

### Short overview of regulated evolutionarily conserved medaka genes, which are also differentially expressed in diverse human cancers

Differentially expressed evolutionarily conserved medaka genes, which were uncovered in Cisplatin- and Trametinib-treated medaka compared to solvent-treated medaka (see 3.1. and 3.2.) were screened if they are also regulated in human melanoma and in other types of human cancers or if they are regulated in a more general context of different human signaling pathways. For identification of these consistently regulated genes the Gene Set Enrichment Analysis (GSEA) software (http://software.broadinstitute.org/gsea/index.jsp) were used (http://software.broadinstitute.org/gsea/index.jsp).

In the context of melanoma and melanoma associated pathways, 10 differentially expressed medaka genes uncovered in the above presented different expression profiles (see 3.1.1., 3.1.2., 3.2.1. and 3.2.2.) were identified to be also regulated in human melanoma and melanoma associated pathways, in particular the genes *DUSP4*, *DUSP6*, *GADD45G*, *IL1R2* (interleukin-1 receptor type 2), *MAP3K8* (mitogen-activated protein kinase kinase kinase 8), *MDM2*, *MKNK2*, *NR4A1*, *PMM1* and *TNF* (tumor necrosis factor) (Table S12). Particularly, the important MAPK pathway, which is a central regulator of melanoma growth showed overlapping features. The WNT/β-catenin signaling pathway, which is often regulated in advanced melanoma, was not found to be regulated in our study, probably due to the early stage of melanoma development represented by our model.

A selection of human gene sets of different cancer types was compared with regulated medaka genes. In particular, comparison to neural tumors revealed eight consistently regulated genes (*ABCC3*, *CUTC*, *ETV4*, *MDM2*, *PHF23*, *PTAFR*, *RNF135*, *SHC3*) (Table S13).

Differentially expressed medaka genes were also compared to human gene sets and signaling pathways, which are not primarily cancer pathways but maybe associated with cancer-related processes (*e.g.*, apoptosis, inflammation, DNA repair, immune-system associated processes) could be detected (Table S14).

## Conclusions

The treatment of juvenile medaka with anti-cancer drugs over a short period of seven days led to considerable changes of the gene expression signature. Through comparisons with non-treated and sham treated fish, we could separate drug-induced side-effects from effects specific for the melanoma fish. For instance, the upregulation of several members of the solute carrier family in cisplatin-treated tg fish was interpreted as a reflection of the general drug-metabolizing processes. Drug-induced expression changes in cisplatin- and trametinib-treated medaka define treatment-induced expression changes in the TTDS. At the same time, the downregulation of *racgap1*, *gmppb* and *gmds* provide strong evidence for a cisplatin-induced suppression of the proliferative and transcriptionally active state of the melanoma tumors and their uncontrolled growth in tg medaka. Trametinib treatment elicited pro-inflammatory and pro-apoptotic expression changes as evidenced by the enhanced expression of genes of the complement system in both wt and tg fish. Reduced expression of genes associated with cellular processes and inhibited protein synthesis was observed in trametinib-treated wt medaka. Treated tg fish differentially expressed several genes enriched in the `MAPK signaling pathway` suggesting a shift from the transformed phenotype to a less malignant phenotype of the tg medaka. We identified only one common gene in tg medaka for both treatments. This result can be expected because these two drugs affect different pathways in cancer cells and their highly complex expression networks. The current study was a first line drug screen important to prove the feasibility of anti-cancer drug screens in the transgenic medaka model. For further determination of a set of common TTDS genes, additional studies are needed. These data will be useful to refine the current TDS set of 365 genes that has been shown to be a useful readout for drug treatment in the transgenic medaka melanoma model.
